# Wee Kinases, Big Impact: Wee and Myt Kinases as Critical Regulators of Meiotic Progression

**DOI:** 10.3390/jdb14030029

**Published:** 2026-07-01

**Authors:** Shannon Pfeiffer, Lourds M. Fernando, Anna K. Allen, Aimee Jaramillo-Lambert

**Affiliations:** 1 Department of Biological Sciences, University of Delaware, Newark, DE 19716, USA; scpfeiff@udel.edu; 2 Department of Developmental Neurobiology, St. Jude’s Children’s Research Hospital, Memphis, TN 38105, USA; lourds.fernando@stjude.org; 3 Department of Biology, Howard University, Washington, DC 20059, USA; anna.allen@howard.edu

**Keywords:** Wee1, Myt1, meiosis, cell cycle, G2/M

## Abstract

Regulation of the cell cycle is critical for maintaining genomic integrity. Therefore, cells have adapted several mechanisms to ensure that cell cycle events occur in a precise order. Some mechanisms regulate cell cycle progression by inhibiting cell cycle drivers, cyclin-dependent kinases (CDKs). The Wee1/Myt1 family of kinases regulate the G2-to-M phase transition by phosphorylating and inactivating Cdk1. Investigations of Wee1/Myt1 have mainly focused on its regulation of mitosis; the role of Wee1/Myt1 kinases in the meiotic cell cycle is less well understood. However, misregulation of Wee1/Myt1 during meiosis can have a range of fertility consequences from mild to severe, including human fertilization failure and infertility. Studies from several organisms reveal that the meiotic functions of Wee1/Myt1 kinases differ from mitosis depending on the species and sex. Here, we review how Wee1/Myt1 kinases regulate cell cycle progression in meiosis across species. We highlight current knowledge of Wee1/Myt1 in meiosis and discuss unanswered questions and new directions to advance the field of meiosis and reproduction. Understanding the molecular and cellular functions of Wee1/Myt1 homologs in these various systems may contribute to the discovery of the mechanisms underlying human infertility cases, better diagnoses, and clinical treatments.

## 1. Introduction

**Figure 1:**
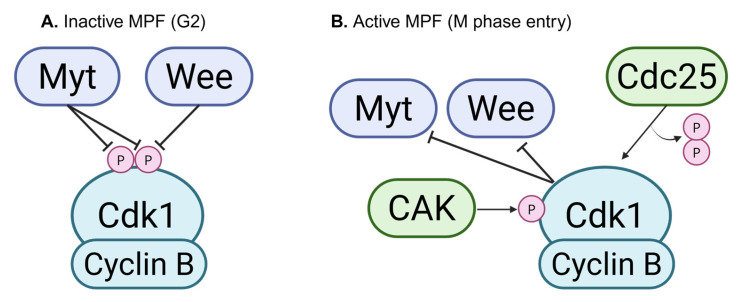
Cdk1/Cyclin B regulation at the G2/M transition. (**A**) Model of Wee1/Myt1 inhibition of Cdk1. Myt kinases are typically dual-inhibitory kinases that phosphorylate Cdk1 at residues Thr 14 and Tyr 15, while Wee kinases typically phosphorylate Tyr 15 only. These phosphorylations inhibit the Cdk1/Cyclin B complex in G2. (**B**) Termination if Wee/Myt inhibition at M phase entry. Cdc25 phosphatase removes pThr 14 and pTyr 15, and Cdk1/Cyclin B is activated by the CDK-activating kinase (CAK). Once active, Cdk1 inhibits Wee/Myt. Created in BioRender. Pfeiffer (2026) https://app.biorender.com/illustrations/654bb02c23b66c90d8fa9c03 (accessed on 7 April 2026).

During the cell cycle, regulatory checkpoints ensure that the appropriate processes occur at the correct time. Progression through the cell cycle is primarily driven by cyclin/cyclin-dependent kinase (CDK) complexes. Different cyclins are expressed at specific cell cycle stages and pair with specific partner CDKs to promote progression through that stage. Since cyclin/CDK complexes are the major drivers of cell cycle progression, certain checkpoints downregulate or inhibit these complexes to prevent premature cell cycle progression. One example occurs at the G2/M (M can refer to both mitosis and meiosis) transition. M phase entry is driven by the Cdk1/Cyclin B complex, also known as maturation-promoting factor (MPF). To ensure that M phase entry occurs only at the appropriate time, MPF regulation is critical. Prior to M phase entry, MPF is inhibited via phosphorylation of Cdk1 by Wee/Myt family kinases. This mechanism prevents cells from entering M phase prematurely in both mitosis and meiosis across eukaryotes (Figure 1). At the G2/M transition, Wee/Myt inhibition is overcome by the counteracting phosphatase Cdc25, which removes the inhibitory phosphorylation from Cdk1 [1,2]. Cdk1 is then activated by the CDK-activating complex (CAK) [3,4]. Once active, Cdk1 can in turn inhibit Wee/Myt, shutting down the inhibitor and committing the cell to M phase entry (Figure 1). There are numerous reviews in the literature that focus on the role of the Wee/Myt kinases during mitosis and specifically in cancer [5,6,7,8]; this review will instead focus on the role of this family of kinases in the specialized cell cycle of meiosis. We summarize the current literature on Wee/Myt kinase regulation during meiosis across a variety of organisms.

## 2. Yeast

### 2.1. Overview

In this section we will review the meiotic functions of Wee1 in both the fission yeast *Schizosaccharomyces pombe* and the budding yeast *Saccharomyces cerevisiae*. Pioneering work in the 1970–1980s discovered Wee1 as a protein required for cell growth and division [1,9,10,11,12,13]. Like the other organisms discussed in this review, Wee1 of both fission and budding yeast is a kinase that phosphorylates a particular tyrosine of CDK1 [14,15]. *S. pombe* Wee1 phosphorylates Tyr 15 of Cdc2 (*S. pombe* CDK1), which inhibits Cdc2 complexed with B-type cyclins preventing entry into mitosis [15]. In the mitotic cell cycle *S. pombe* Cdc2 phosphorylation is maintained throughout interphase until the onset of mitosis when Cdc25 and Pyp3 phosphatases remove Tyr 15 phosphorylation. This allows for full activation of Cdc2 and progression of the cell cycle [15,16,17,18]. In *S. cerevisiae* Swe1 (homolog of fission yeast Wee1) phosphorylates Tyr 19 of Cdc28 (analogous to Cdc2) [14]. Swe1 negatively regulates Cdc28-Cyclin B complexes; however, unlike *S. pombe* and other eukaryotes, Swe1 does not determine cell cycle timing. Rather, Swe1-mediated phosphorylation delays nuclear division when there are defects in bud formation [14].

### 2.2. Expression and Localization

*S. pombe* Wee1 is an 877-amino-acid protein with a molecular weight of 105 kilodalton (kDa) [13,19]. During meiosis both Wee1 mRNA and protein levels accumulate after meiotic induction with maximum levels reached at the initiation of premeiotic DNA replication. After DNA replication, Wee1 protein levels decrease and are undetectable by the meiotic M phase [19]. Downregulation of Wee1 expression is transcriptionally controlled by Mei4, which also simultaneously activates transcription of the Cdc25 phosphatase that activates Cdc2 [20]. Wee1 protein localization has not been determined in cells undergoing meiosis. However, Wee1 exhibits dynamic localization during mitosis localizing to the nuclear face of the spindle pole body (SPB, analogous to the metazoan centrosome) during the G2/M transition and then disappears during spindle assembly [21]. Masuda et al. [21] hypothesized that SPB Wee1 accumulation helps regulate mitotic spindle assembly. Perhaps Wee1 plays a similar function in meiosis.

In *S. cerevisiae* Swe1 is an 819-amino-acid, 92 kDa protein [14]. Swe1 mRNA expression increases ~10-fold after meiotic induction at a time point that corresponds to pachytene of meiotic prophase I [22]. Swe1 protein levels temporarily decrease after meiotic induction before accumulating and peaking at pachytene. After pachytene, Swe1 protein levels rapidly decline with very little protein remaining at the first meiotic division [22]. As with *S. pombe* Wee1, the meiotic localization of Swe1 has not been determined. In mitosis, Swe1 localizes to the bud neck (Swe1-GFP overexpressed from a multicopy vector, Swe1-GFP at endogenous levels was undetectable) [23].

### 2.3. Function

In both fission and budding yeast meiosis, Wee1 is present (Table 1), but does not appear to regulate cell cycle timing. Genetic analysis of loss-of-function mutants of either *S. pombe wee1* or *S. cerevisiae swe1* found that the mutants proceed through meiosis normally [22,24]. In *S. pombe* Wee1 may play a role in the premeiotic DNA replication checkpoint. Meiotic cells treated with the DNA replication inhibitor hydroxyurea (HU) arrest and the checkpoint maintains Cdc2 phosphorylation. However, meiotic cells homozygous for a temperature-sensitive *wee1* allele and a deleted *mik1* allele (an additional kinase responsible for phosphorylating Cdc2) are still capable of arresting in the presence of HU [25,26]. In this study the authors hypothesize that an additional checkpoint, independent of Cdc2 phosphorylation, exists, but this pathway has not yet been elucidated.

In budding yeast Swe1 functions as part of the meiotic recombination checkpoint. When recombination intermediates are present or homologous chromosomes are not properly synapsed, meiotic cells arrest at the pachytene stage, which is when homologous chromosomes should be fully synapsed [22,27]. Deletion of *swe1* alleviates this arrest with cells nearly reaching wild-type levels of nuclear division, although division is delayed [22]. The Swe1-mediated pachytene checkpoint arrest is driven through Tyr 19 phosphorylation of Cdc28, as cells with a non-phosphorylatable form of Cdc28 (*cdc28-AF*) are still capable of meiotic nuclear divisions when chromosome synapsis is defective [22]. Regulation of Swe1 activity in response to checkpoint activation appears to be at the level of post-translational modification. Swe1 mRNA levels are similar in both the wild type and in cells with pachytene checkpoint activation [22]. However, Swe1 protein levels continue to accumulate and reach a maximum level that is four-fold higher than the wild type. In addition, Swe1 becomes hyperphosphorylated when the pachytene checkpoint is triggered [22]. Thus, accumulation of Swe1 early in meiosis positions cells to quickly respond to recombination or synapsis defects, which then trigger phosphorylation of Swe1 to presumably increase the activity level of Swe1 to prevent the cells from moving through the nuclear divisions with defects.

**Table 1:** Summary of expression and localization data for Wee family kinases that play a known role in meiotic regulation in indicated species.

Species	Wee Family Kinases	Wee Kinases with Known Meiotic Roles	Subcellular Localization During Meiosis
*S. pombe*	Wee1	Wee1	Expressed during meiosis, localization unknown [19].
*S. cerevisiae*	Swe1	Swe1	Expressed during meiosis, localization unknown [14].
*D. melanogaster*	dMyt1, dWee1	dMyt1	Localizes to intracellular junctions, Golgi, meiotic spindle, and fusome bridges in spermatocytes [28]. Unknown in oocytes.
*C. elegans*	WEE-1.1, WEE-1.3	WEE-1.3	Perinuclear during meiotic prophase [29,30].
*C. hookeri*	Wee1a, Wee1b	Wee1a, Wee1b	RNA expressed in gonad tissue [31].
*A. pectinifera*	Myt1, Wee1	Myt1	Expressed in oocytes [32]. Unknown in spermatocytes.
*X. laevis*	Myt1, Wee1a, Wee1b	Myt1	Expressed in oocytes [33,34]. Unknown in spermatocytes.
*A. testudineus*	Myt1, Wee1	Myt1	Expressed in oocytes [35]. Unknown in spermatocytes.
*M. musculus*	PKMyt1, Wee1, Wee2	PKMyt1	Cytoplasmic in oocytes [36].
		Wee1	Perinuclear in prophase spermatocytes [37].
		Wee2	Nuclear in prophase oocytes, exported prior to GVBD [36].
*S. scrofa*	PKMyt1, Wee1a, Wee1b	PKMyt1	Unknown during meiosis.
		Wee1a	Unknown during meiosis.
		Wee1b	Nuclear in oocytes [38].
*H. sapiens*	PKMyt1, Wee1, Wee2	PKMYT1	Expressed in spermatocytes and oocytes.
		WEE1	Expressed in spermatocytes [39] and oocytes.
		WEE2	Expressed in oocytes [40].

## 3. *C. elegans*

### 3.1. Overview

The nematode *Caenorhabditis elegans* has two Wee1/Myt1 kinase family members, *wee-1.1* and *wee-1.3* (*wee-1.2* is a pseudogene and will not be discussed in this review). Since *wee-1.1* expression is restricted to the embryo during the 12-to-16 cell stage [41], *wee-1.3* is the primary Wee/Myt kinase family member in *C. elegans*. WEE-1.3 is a dual-specificity kinase that inhibits the Cdk1 (*C. elegans* CDK-1)/Cyclin B complex (also referred to as maturation-promoting factor, or MPF) by phosphorylating CDK-1 at residues Thr 14 and Tyr 15. This mechanism prevents cells from entering M phase prematurely. Activation of CDK-1/Cyclin B requires both dephosphorylation of pThr 14 and pTyr 15 by Cdc25 (*C. elegans* CDC-25), the counteracting phosphatase to WEE-1.3 kinase [42], and phosphorylation of Thr 161 by CDK-7, the CDK-activating kinase (CAK) in worms [4]. Once a threshold level of active CDK-1 is reached, WEE-1.3 inhibition is overcome and the cell cycle resumes.

### 3.2. Expression and Localization

The *wee-1.3* gene encodes a 677-amino-acid, 76.9 kDa protein with a transmembrane domain which separates the conserved serine/threonine kinase domain from a non-conserved C-terminal domain of uncharacterized function [43]. *wee-1.3* is expressed in the *C. elegans* germline during oogenesis and spermatogenesis and plays essential, but different, roles regulating the timing of female and male meiosis.

During oogenesis, WEE-1.3 is expressed in a perinuclear pattern throughout the *C. elegans* germline [30]. It is also visible as cytoplasmic puncta. At diakinesis, WEE-1.3 coats the chromosomes [29]. During spermatogenesis, WEE-1.3 displays a similar perinuclear expression throughout the male germline. It is also visible in the cytoplasm. Notably, WEE-1.3 is also visible at the centrosomes of metaphase I spermatocytes (unpublished data, Jaramillo-Lambert lab). As in most animals, including humans, *C. elegans* oocytes lack centrosomes, which are supplied by the sperm upon fertilization [44,45]. Hence, it is impossible for WEE-1.3 to localize to centrosomes in oocytes.

### 3.3. Function

While WEE-1.3 inhibits CDK-1/Cyclin B in both oogenesis and spermatogenesis, the precise mechanisms differ between the two. This may depend on the identity of the B cyclin and the timing of M phase entry, among other factors. Notably, *C. elegans* have four B-type cyclins [46,47]. Of these, cyclins B1 and B3 are essential for meiosis. Cyclin B1 (CYB-1) is required for spermatogenesis, but is dispensable during oogenesis [48]. Cyclin B3 (CYB-3) is required for oogenesis [49], while knockdown of *cyb-3* does not affect spermatogenesis [48]. This suggests that, while WEE-1.3 inhibits CDK-1/Cyclin B in both spermatogenesis and oogenesis, the presence of different B cyclins in the complex during each type of meiosis could lead to different downstream effects.

Another key difference affecting WEE-1.3 regulation during oogenesis compared to spermatogenesis is the timing of meiotic entry. *C. elegans* oocytes arrest late in prophase I during diakinesis. WEE-1.3 maintains this arrest by inhibiting MPF. Release from meiotic arrest is precipitated by major sperm protein (MSP) signaling provided by sperm [50]. Upon release from arrest, the now-active MPF complex facilitates the key events of oocyte maturation, such as nuclear envelope breakdown (NEBD), chromosome compaction, and cytoskeletal changes including cortical actin rearrangement and spindle assembly [51]. Knockdown of *wee-1.3* induces premature oocyte maturation, including premature NEBD, defects in chromosome condensation, and early rearrangements of cortical actin and spindle microtubules [51]. As a consequence of undergoing premature oocyte maturation, *wee-1.3-*depleted oocytes also ectopically express many egg activation and eggshell proteins, such as MBK-2, CHS-1, and CBD-1 [29]. Since these oocytes are fertilization-incompetent, it is likely that premature expression of eggshell proteins causes eggshell formation pre-fertilization that physically blocks sperm entry. An RNAi suppressor screen identified 44 genes that interact with *wee-1.3* during oogenesis [29]. In addition to cell cycle regulators, the screen identified other classes of proteins including proteasome components and nuclear import proteins. Depletion of the proteasome component and *wee-1.3* interactor *rpn-6.1* disrupts normal WEE-1.3 localization, resulting in WEE-1.3 localizing to the nucleus of developing oocytes, rather than the nuclear membrane [52]. Further work is needed to uncover the mechanisms by which each of these genes interact with WEE-1.3 during oogenesis.

In *C. elegans* spermatogenesis, missense mutations in a four-residue region in the C-terminal domain of WEE-1.3 prevent spermatocytes from entering M phase, resulting in sterility [43]. Notably, the phenotypes of these mutations are spermatogenesis-specific, as mitosis and oogenesis are not affected. This indicates that in *C. elegans* the C-terminal domain of yet-uncharacterized function is required for WEE-1.3 to regulate spermatogenesis. Proper localization of the C-terminal domain is crucial for its role in spermatogenesis, as removal of the transmembrane domain in WEE-1.3 suppresses the spermatogenesis phenotype in worms with the C-terminal missense mutations [43]. While not genetically conserved with *C. elegans*, the C-terminal domain of Myt1 in *Xenopus* plays a regulatory function during oocyte maturation [53]. Perhaps the C-terminal domain plays a similar role in *C. elegans* spermatogenesis. It is also possible that the precise mechanism of WEE-1.3 regulation via the C-terminal domain is novel. Although regulatory mechanisms may differ between species, *C. elegans* is not the only species that exhibits spermatogenesis-specific WEE-1.3 regulation. *Drosophila* Myt1 is required to maintain proper meiotic spindle architecture and centrosome number during spermatogenesis, and loss of Myt1 causes infertility in males, but not females [28]. Preliminary data indicates that WEE-1.3 localizes to centrosomes in prometaphase spermatocytes (unpublished data, Jaramillo-Lambert lab), suggesting possible similarities between flies and worms in spermatogenesis regulation.

Many other organisms have multiple Wee/Myt family kinases, with different members playing different tissue-specific roles. *C. elegans* is unique in that WEE-1.3 is the primary Wee/Myt family kinase member. This single kinase must function in multiple different ways to differentially regulate different types of cell division, including mitosis, oogenesis, and spermatogenesis. This may be achieved through regulatory domains in WEE-1.3, the specific B cyclin in the CDK-1/Cyclin B complex, CDC-25 phosphatase type and activity, and different feedback systems depending on the timing of M phase entry in different cell types. Much work is needed to elucidate these mechanisms. Understanding how this single, multi-functional Wee kinase can differentially regulate cell divisions will improve our understanding of how different Wee/Myt kinases perform specific functions in other animals.

## 4. *Drosophila*

### 4.1. Overview

In *Drosophila* there are two Wee1-related Cdk1 inhibitory kinases, dWee1 nuclear kinase and dMyt1 membrane-associated kinase, which are crucial in maintaining cell cycle arrest during mitosis and meiosis [28,54,55,56]. dWee1 mono-phosphorylates Cdk1 on Tyr 15, while dMyt1 is a dual-specificity kinase that phosphorylates both the Thr 14 and Tyr 15 residues of Cdk1 [57,58,59]. Maternally provided dWee1 is essential for Cdk1 regulation during the rapid mitotic cell divisions which occur during early embryogenesis. However, zygotic dWee1 is not required for subsequent embryonic development, with zygotic *wee1* mutants being viable and having no observable developmental defects [55,60,61]. dMyt1 regulates Cdk1 throughout development and gametogenesis, and has been implicated in wing imaginal disc development, germ cell proliferation, and spermatogenesis [28,55]. We will limit our discussion to dMyt1 because of its exclusive involvement in *Drosophila* meiosis.

### 4.2. Expression and Localization

*Drosophila* Myt1 (dMyt1) is a 533-amino-acid-long, 61.2 kDa protein with a conserved kinase domain, potential transmembrane domain, and a putative C-terminal Cyclin B binding motif [55]. The dMyt1 kinase domain shares 47–49% amino acid sequence identity with the *Xenopus* and Human Myt1 homologs, but only 31% identity with the nuclear dWee1 kinase domain [57]. A lack of dMyt1 antibodies has hindered its spatiotemporal study, and antibodies that recognize other Myt1 kinases fail to recognize dMyt1 [58]. Currently, the available localization data are from studies using recombinantly overexpressed dMyt1/V5 in cultured *Drosophila* S2 cells and transgenic Gal4-inducible UASp-*EGFP-Myt1* strains [28,58]. dMyt1/V5 expression was observed as discrete cytoplasmic puncta which are partially co-localized with a known Golgi marker [58]. A role for dMyt1 in mitotic Golgi behavior was confirmed when depletion of dMyt1 via RNAi in S2 cells resulted in incomplete Golgi fragmentation [58]. The EGFP-Myt1 strain further confirmed both Golgi and endoplasmic reticulum expression of Myt1 during male meiosis [28]. In pre-meiotic G2 arrested polar spermatocytes, EGFP-Myt1 is mainly cytoplasmic but exhibits enrichment at the intercellular junctions and partial co-localization with the fusome bridges interconnecting the spermatocytes [28]. Fusomes are specialized, membranous structures that interconnect developing germ cell cysts (both ovarian and spermatogenic) and function in coordinating cyst cell division, intercellular communication, and, in female germline cysts, oocyte determination [62]. The EGFP-Myt1 intercellular junction enrichment and co-localization with fusome bridges goes away in the subsequent apolar spermatocyte stage where staining is just cytoplasmic, and then in mature spermatocytes there is an appearance of EGFP-Myt1 cytoplasmic puncta resembling Golgi stacks [28]. Once the spermatocytes enter meiosis, EGFP-Myt1 is observed on the meiotic spindle during metaphase I before becoming cytoplasmic again post-meiosis [28]. There have been no published reports utilizing the EGFP-Myt1 transgenic strain to elucidate expression of dMyt1 during oogenesis.

### 4.3. Function

Phenotypic and genetic analyses of dMyt1 have shown its conserved role in regulating Cdk1 in somatic and reproductive tissues during the G2 phase cell cycle arrest in mitosis and meiosis [54,55]. Canonical dMyt1 function is crucial in different stages of *Drosophila* gametogenesis. During *Drosophila* spermatogenesis, the sperm undergo a prolonged premeiotic cell cycle arrest for nearly 90 h in the G2 phase before entering meiosis, allowing time for the sperm to synthesize cellular components needed for subsequent development [28,63,64]. The spermatogenesis arrest is maintained in two ways: (1) by downregulation of Cyclin B production until the late premeiotic G2 phase, and (2) inactivation of Cdk1/Cyclin A and Cdk1/Cyclin B complexes by the dMyt1 kinase [28,55]. In contrast, *Drosophila* oocytes do not undergo prolonged premeiotic cell cycle arrest. Rather, the oocytes arrest at two different stages of meiosis I, first at prophase I and then at metaphase I. Both oocyte arrests are thought to be maintained by the dMyt1-dependent inactivation of the Cdk1 partner of the Cdk1/Cyclin B complex [65,66].

A loss-of-function *myt1* mutant in *Drosophila* is male sterile but not female sterile [28,55]. Phenotypic analysis of these male *myt1* mutants revealed compromised fusome integrity in primary spermatocytes, aberrantly decondensed chromosomes, fragmented nucleoli, and premature centriole disengagement in immature spermatocytes resulting in multipolar meiotic spindles [28]. The fusomes are important for the proper distribution of centrosomes and other molecular factors among the cells in the germline cysts and are essential for both male and female gametogenesis [62]. The fusome sequesters Cdk1/Cyclin A phosphoinactivated by dMyt1 during premeiotic G2 phase arrest in spermatocytes and this is important to maintain the fusome structure [28]. Loss of dMyt1 precociously activates Cdk1/Cyclin A in a Cdc25 phosphatase-dependent manner and destabilizes fusomes [28]. Loss of dMyt1 and subsequent precocious Cdk1/Cyclin A activity could then activate *Drosophila* Polo-like kinase (Plk1) which results in the premature centriole disengagement phenotype. Prematurely activated Plk1 initiates centriole disengagement before meiosis I, ultimately causing multipolar spindle formation leading to aneuploidy which can contribute to male sterility [28]. siRNA depletion of Plk1 was sufficient to rescue the *myt1* mutant premature centriole disengagement phenotype; however, it does not rescue the *myt1* mutant fusome defects. The mechanisms by which Cdk1/Cyclin A maintain fusome integrity have not yet been elucidated and there is a big gap in knowledge on how Plk1 regulates centriole disengagement. Perhaps dMyt1 investigations may contribute to elucidating these mechanisms. Interestingly, ectopic expression of dWee1 could rescue the *myt1* mutant premature centriole disengagement, multipolar spindles, and mutant sterility, but could not rescue the fusome defects [28]. Therefore, it has been hypothesized that the sterility observed upon Myt1 depletion is not due to the altered timing of the G2/M transition in *Drosophila* spermatocytes but is rather a consequence of premature activation of Cdk1/Cyclin A complex that controls fusome integrity and centriole engagement during premeiotic G2 phase arrest [28].

Although *myt1* mutation did not cause sterility in *Drosophila* females, a high incidence of embryonic lethality confirms that dMyt1 also plays an important role in oogenesis [55]. Like male germ cells, oocytes also grow in interconnected germline cysts connected by fusome intercellular bridges; however, in oogenesis only one cell in the 16-cell cyst develops to become an oocyte [67]. The other 15 cells differentiate into nurse cells that enrich the oocyte with mRNA, proteins and nutrients needed for oocyte development and early embryogenesis after fertilization [67]. Unlike spermatocytes, oocytes do not undergo prolonged premeiotic G2 phase cell cycle arrest. Rather, they arrest at meiotic prophase I until ovulation then arrest for the second time in metaphase I [65,66,67]. Oocyte cell cycle arrests are maintained mainly by proteasomal degradation of Cyclin B targeted by APC/C E3 ligase thus keeping Cdk1 inactive [68]. Involvement of dMyt1 in maintaining oocyte meiotic arrest is therefore not essential in *Drosophila*. This is interesting because dMyt1 homologs in other organisms, including mammals, are the major inhibitor of Cdk1/Cyclin B during cell cycle arrest in meiosis and mitosis.

Nevertheless, loss of dMyt1 results in chromosome segregation defects in *Drosophila* oocytes leading to high rates of embryonic lethality due to aneuploidy [55]. Oocytes lacking dMyt1 exhibit an increased frequency of chromosome non-disjunction (NDJ) events, specifically of the X and 4th chromosomes, resulting in defective oocytes and non-viable embryos [55]. The NDJ defects in oocytes may be caused by faulty regulation of kinetochore proteins or proteins involved in sister chromatid adhesion, such as cohesins, during metaphase I arrest. The lack of information that connects dMyt1 to kinetochore proteins or cohesin regulation blocks one from building a more informed hypothesis; therefore, much work is needed to address dMyt1 interacting proteins in *Drosophila* meiosis. Perhaps this might call for a genetic screen similar to the one done with the *C. elegans* ortholog of dMyt1, WEE-1.3 [29]. Furthermore, studies should focus on other Cdks and cyclins in *Drosophila* that are involved in meiosis and investigate whether dMyt1 targets other Cdks, such as Cdk2 associated with Cyclin E.

Gametogenesis involves both mitosis to amplify germ cell numbers and meiosis to generate the haploid gametes. dMyt1 has been demonstrated to function in mitotic proliferation during both spermatogenesis and oogenesis [55]. Loss of dMyt1 results in over-proliferation of germ cells undergoing mitosis prior to entering meiosis in both male and female germlines. The mitotic index of germline stem cells (GSCs) in female germlines increase 20-fold in the absence of dMyt1 resulting in smaller-than-usual GSCs [55]. Male germlines of *myt1* mutants show secondary spermatogonia failing to enter meiosis on time resulting in an extra round of mitosis [55]. Moreover, loss of dMyt1 also contributes to ectopic cell division of germline-associated somatic cells.

## 5. *Xenopus*

### 5.1. Overview

*Xenopus* has three members of the Wee1/Myt1 kinase family—Wee1A, Wee1B, and Myt1 [33,69,70]. The three members of the *Xenopus* Wee1/Myt1 kinases inhibit Cdk1 (Cdc2) activity through phosphorylation. Wee1A and Wee1B phosphorylate Tyr 15 while Myt1 phosphorylates both Thr 14 and Tyr 15 of Cdk1 [33,69,70]. During M phase of mitosis and meiosis, the Cdc25 phosphatase dephosphorylates Thr 14 and Tyr 15 allowing for activation of the Cdk1/Cyclin B complex and cell cycle entry [71,72]. Active Cdk1 inhibits Wee1/Myt1 kinases and activates Cdc25 phosphatase via phosphorylation. These feedback loops create a switch-like activation system for Cdk1/Cyclin B [73].

### 5.2. Expression and Localization

Wee1A is the shorter of the two *Xenopus* Wee1 isoforms consisting of 555 amino acids and a molecular weight of 62 kDa [69]. Wee1B has an additional four amino acids (559 amino acids) with a molecular weight of 67 kDa [70]. Myt1 is a 548-amino-acid protein with a molecular weight of 62 kDa [33]. All three consist of three main domains including the N-terminal regulatory region (NRD), a central kinase domain (KD), and a C-terminal regulatory region (CRD) [70,74,75].

Wee1A mRNA is first expressed at the onset of meiosis II and remains at constant levels until gastrulation [72,76]. Low levels of Wee1A protein were detected in early stages of meiotic prophase I; however, Wee1A protein is no longer detected in late prophase I arrested oocytes, nor is it detected in oocytes undergoing germinal vesical breakdown (GVBD) during oocyte maturation [72]. Wee1A protein is found in mature oocytes approximately one hour post-GVBD and in early embryos then gradually disappears during gastrulation. Wee1B mRNA is present in oocytes, pregastrula embryos, and postgastrula embryos, but Wee1B protein is only detectable during gastrulation through the tailbud stages of embryogenesis [70]. Due to the different expression levels of the two Wee1 isoforms, it has been proposed that Wee1A is a maternal isoform required for rapid cell cycles prior to gastrulation and Wee1B is the zygotic isoform required for the longer cell cycles after the midblastula transition [70].

Myt1 protein is present in immature oocytes arrested at prophase I [33,34]. Myt1 protein is membrane-associated with a predicted transmembrane domain, while Wee1A and Wee1B are found in the cytosol [33,69].

### 5.3. Function

In *Xenopus*, only Myt1 is found in immature oocytes. Wee1A and Wee1B are not present in immature prophase I-arrested oocytes and are not involved in the regulation of pre-MPF in oocytes. Furthermore, it is crucial that Wee1A and Wee1B proteins are absent to ensure that DNA replication does not occur between meiosis I and meiosis II. Ectopic expression of Wee1 caused oocytes to enter interphase and DNA synthesis after meiosis I [72,73].

Prior to meiotic entry, a stockpile of MPF in an inactive state (pre-MPF) accumulates throughout oogenesis [77]. At the same time, Cyclin B is slowly synthesized. Newly formed Cdk1/Cyclin B complexes are immediately inhibited by Myt1, which arrests oocytes at the G2/M boundary of meiosis I [71,78]. Loss of Myt1 through antibody knock-down causes premature meiosis I entry [72]. In the wild type, meiotic resumption is triggered by the hormone progesterone [79]. Gaffre et al. proposed a model whereby a balance of Myt1 activity and Cyclin B synthesis is critical for the transition between prophase arrest and meiotic entry. They found that Myt1 phosphorylated newly formed Cdk1-Cyclin B complexes [71]. However, they propose that the increased rate of Cyclin B synthesis in response to progesterone is sufficient to allow some Cdk1-Cyclin B complexes to “escape” the inhibitory phosphorylations of Myt1 [80,81]. In turn, Cdk1/Cyclin B complexes can now phosphorylate and inhibit Myt1. This occurs before activation of Cdc25 phosphatase, which requires both Cdk1/Cyclin B complexes and the MAPK pathway to become fully activated [71]. Together, these events create an auto-amplification loop that allows oocytes to enter meiosis.

Both the NRD and CRD are important for *Xenopus* Myt1 regulation. Multiple sites in the Myt1 CRD are phosphorylated by Cdk1/Cyclin B, p90rsk, Cdk-RINGO, and Plk1 and are thought to be involved in the inactivation of Myt1 [34,71,82]. In the NRD, Myt1 autophosphorylation of Ser 66 and Ser 76 and Cdk1-mediated phosphorylation of Thr 11, Thr 16, Ser 32, Ser 79, and Thr 89 all negatively regulate Myt1 activity [74,83]. In addition to negative regulation, a PAYF motif (a short-conserved sequence of _21_(Pro)-Ala-Tyr-(Phe)_24_) in the NRD of Myt1 is required to positively impact Myt1 activity [74]. When Myt1 mRNA containing mutations in the PAYF motif were injected into oocytes, Cdk1 phosphorylation was greatly reduced and the oocytes showed reduced inhibition of GVBD [74]. Taken together, the regulation of Myt1, Cdk1/Cyclin B, and meiotic resumption is a complex process that will require additional studies to determine the exact mechanisms of regulation.

## 6. Mammals

### 6.1. Overview

Mammals have three Wee kinase family members: Wee1, Wee2, and PKMyt1. Since much of our knowledge of Wee family kinases comes from research done in mice, this section will primarily focus on mouse Wee1, Wee2, and PKMyt1 but will also include information about these kinases in other mammals, including pigs and humans. Wee2 and PKMyt1 are both active during oogenesis with Wee2 being the primary Wee kinase that regulates oogenesis in mammals, including mice, cows, pigs, and macaques [38,84,85]. While Wee kinases have been primarily studied in oogenesis, Wee1 is also known to regulate mammalian spermatogenesis [39,86,87].

### 6.2. Expression and Localization

Mammalian Wee1, Wee2, and PKMyt1 kinases all function to inhibit Cdk1. Wee1 and Wee2 phosphorylate Cdk1 at Tyr 15, while PKMyt1 is a dual-specificity kinase that phosphorylates Thr 14 and Tyr 15. Each of these kinases are highly conserved in mammals. Wee2 has a nuclear export signal (NES) and nuclear localization signal (NLS) that is conserved in mammals including mice and pigs [36,88,89]. During mouse oogenesis, Wee2 localization is dynamic. Microinjection of GFP-Wee2 mRNA into mouse oocytes shows that Wee2 is nuclear in arrested oocytes, but is exported into the cytoplasm upon meiotic resumption, prior to germinal vesicle breakdown (GVBD) [36]. Wee2 nuclear export coincides with Cdc25B and Cdk1/Cylin B import into the nucleus, 15–20 min before GVBD in mice [36,90]. This exclusion of Wee2 from the nucleus sequesters it from nuclear MPF. Cdc25B then activates MPF in the nucleus by dephosphorylating Cdk1. While this process has not been directly observed in pig oocytes, overexpression of nuclear exportin XPO1 causes pig Wee2 to be exported during meiotic arrest, resulting in premature M phase entry [91]. This suggests that pig Wee2 localization dynamics at M phase entry are likely the same as mouse Wee2 and may be conserved in other mammals. In contrast to Wee2, PKMyt1 is cytoplasmic and does not change localization upon meiotic resumption in mouse oocytes [36].

During mouse spermatogenesis, Wee1 is present in the nucleus and cytoplasm of spermatocytes in the early stages of meiotic prophase I (leptotene–pachytene). By diplotene, one of the final stages of prophase I, Wee1 is no longer visible. While the localization of Wee1 during human spermatogenesis is unknown, Wee1 mRNA is expressed in testes [39].

### 6.3. Function

Here, we will discuss the roles of Wee2 and PKMyt1 in oogenesis and Wee1 in spermatogenesis. Similar to other organisms discussed in this review, the timing of M phase entry in mammals differs greatly between oogenesis and spermatogenesis. While spermatocytes develop and enter meiosis without pause, oocytes maintain prolonged arrests between birth, ovulation, and fertilization. The oocyte meiotic arrest in mammals is maintained by keeping Cdk1 inactive during the oocyte growth period. There are two major ways by which MPF is kept inactive in mammals: (1) inactivation of Cdk1 by Wee/Myt kinases, and (2) proteasomal degradation of Cyclin B.

Downregulation of Wee2 via RNAi in mice causes germinal vesicle breakdown, a hallmark of oocyte meiotic maturation, and polar body extrusion, indicating that Wee2 is responsible for maintaining oocyte arrest [88]. One of the major regulators of Wee2 is protein kinase A (PKA), which is activated by high cAMP levels in arrested oocytes [84,92]. PKA activates Wee2 by phosphorylating its Ser 15 residue [92]. This is similar to pig oogenesis, where PKA phosphorylates pig Wee2 at Ser 77 [89]. PKA phosphorylation keeps Wee2 active and able to inhibit Cdk1 during the prolonged oocyte meiotic arrest [92]. During ovulation Cdc25 removes the inhibitory phosphorylations on Cdk1 and oocytes resume meiosis. Wee2 activity on Cdk1 at oocyte meiotic maturation is governed by the active export of Wee2 to the cytoplasm of the oocytes [36].

Upon resumption of meiosis and progression through the meiotic cell cycle, Cdk1 must be inhibited for the oocyte to exit metaphase II [93]. At metaphase II exit, Wee2 is activated through phosphorylation of its Ser 15 residue by Calmodulin-dependent kinase II (CaMK II) [93]. This is the same residue at which PKA phosphorylates Wee2 during meiotic arrest in mice, indicating that Ser 15 is an important regulatory site. CaMKII activation of Wee2 at metaphase II exit allows Wee2 to inhibit Cdk1, promoting timely APC activation. The APC in turn targets Cyclin B for proteasomal degradation, completely terminating MPF signaling once meiosis II has begun [93].

Switching from mouse to humans, recent studies have emerged linking mutations in Wee2 to infertility in women [94,95,96,97]. One whole-genome sequencing study of four women with fertility defects identified homozygous mutations solely in the *Wee2* gene [40]. The mutations that were found in the *Wee2* gene decreased the production of the protein and reduced Wee2 Ser 15 phosphorylation. As a result, Tyr 15 phosphorylation of Cdk1 was reduced [40]. Most importantly, the oocytes from the affected individuals gained fertilization competency upon *Wee2* mRNA introduction [40]. Several studies have identified homozygous and compound heterozygous Wee2 mutations associated with infertility in patients [98,99,100]. In these patients, loss of Wee2 regulation likely causes premature Cdk1 activation leading to production of defective oocytes. The indispensable nature of Wee2 in female fertility makes Wee2 a promising target for contraceptive development. Intriguingly, a high-throughput drug screen identified three Wee2-specific inhibitors as potential candidates for non-hormonal contraceptives [101]. More recently, an in silico screen of FDA-approved compounds identified two drugs that should be explored as candidates [102].

While Wee2 regulates oogenesis, Wee1 is the primary Wee family member responsible for spermatogenesis regulation. One study of 37 male human patients found that transcript levels of key M phase regulators including Wee1, Cdk1, Cyclin B1, Cyclin B2, Cdc25A, and Cdc25C were all downregulated in patients exhibiting spermatogenesis failure [39]. Maintaining proper levels of Wee1 and regulating its activation and inactivation are crucial for sperm production. A study in mice found that loss of Cdc14A results in male subfertility [103]. Cdc14A is a phosphatase that dephosphorylates Wee1 at Ser 123 and Ser 129 leading to Wee1 activation [86]. These Wee1 residues are phosphorylated by Cdk1 to deactivate Wee1 once a threshold level of active Cdk1/Cyclin B is reached at M phase entry. In a similar manner, Cdc14A also dephosphorylates Cdc25A, which is phosphorylated by Cdk1 to further promote Cdk1/Cyclin B activation [86,87]. These findings establish Cdc14A as a critical regulator that counteracts the feedback loops driven by active Cdk1.

In addition to inhibiting Cdk1, Wee1 is also required to inhibit Cdk2, which is associated with S phase regulation, during mammalian spermatogenesis. Mutating Tyr 15 of Cdk2 prevented Wee1 from phosphorylating Cdk2 and resulted in infertility in male mice [87]. Spermatocytes in these mutant mice were arrested at the pachytene stage of prophase I and exhibited defects in homologous chromosome synapsis and unrepaired double strand breaks. An overall reduced number of spermatocytes also indicates that Wee1 regulation of Cdk2 is critical to maintain mitotic proliferation of spermatogonial stem cells [87]. Interestingly, female mice with the same Cdk2 Tyr 15 Ser mutation were fertile, while Cdk2 null mutant females are infertile, indicating that a different Wee family kinase, Wee2 or PKMyt1, is responsible for Cdk2 regulation during oogenesis [87].

## 7. Other Organisms

### 7.1. Climbing Perch (Anabas testudineus)

Climbing perch have two Wee1 orthologs, Wee1 and Wee2 (also referred to as Myt1). Wee2/Myt1 is the oogenesis-specific Wee kinase family member. Wee2/Myt1 is a 553-amino-acid protein with conserved ATP binding and kinase domains. The role of Wee kinases have not been investigated in perch spermatogenesis.

While the subcellular localization of Wee2/Myt1 in *A. testudineus* oocytes is not known, both Wee2/Myt1 transcript and protein are present in both immature and mature oocytes [35]. As in other animals, Wee family kinases inhibit the Cdk1/Cyclin B complex in climbing perch. During oogenesis, oocytes are released from arrest upon exposure to maturation-inducing hormone (MIH) [35]. Release from arrest requires both Wee2/Myt1 inactivation and Cdc25 activation. Phosphorylated levels of both Wee2/Myt1 and Cdc25 increase upon MIH exposure. Myt1 phosphorylation is dependent upon Mos activity, although it remains unclear whether Mos directly phosphorylates Myt1 [35]. Phosphorylation of Cdc25 is independent of Mos1, and the mechanism of Cdc25 activation remains to be elucidated.

### 7.2. Starfish (Asterina pectinifera)

Starfish have two Wee1 family orthologs, Wee1 and Myt1. Of these, only Myt1 is required for oocyte meiosis. Starfish Myt1 is a 565-amino-acid protein that contains a conserved kinase domain. Since Myt1 has only been studied during oocyte meiosis in starfish, and not in spermatocyte meiosis, this section will focus on oogenesis. Although the localization of Myt1 has not been examined in starfish, Western blot data demonstrates that Myt1 is present in oocytes [32].

The function of Myt1 as a Cdk1/Cyclin B inhibitor during oogenesis is conserved in starfish, with Myt1 inhibiting Cdk1 until a threshold level of active Cdk1/Cyclin B is reached. During starfish oogenesis, meiotic entry is triggered by 1-MeAde (1-methyladenine), the oocyte maturation-inducing hormone [104]. Downstream of 1-MeAde signaling, the kinase SGK is activated and ultimately triggers the G2/M transition by simultaneously inhibiting Myt1 and activating Cdc25 [32]. This tilts the balance of Cdk1/Cyclin B in favor of active Cdk1. Once a threshold level of active Cdk1/Cyclin B is reached, feedback loops drive further inactivation of Myt1 and activation of Cdc25 [32]. This mechanism ensures that, once a threshold level of stimulating hormone is detected, the oocyte is irreversibly committed to M phase entry. Further work in the starfish has elucidated threshold-sensing mechanisms that prevent Myt1 from being prematurely inactivated. At sub-threshold levels of 1-MeAde, Myt1 and Cdc25 are initially phosphorylated, causing Myt1 inactivation and Cdc25 activation. However, both proteins are dephosphorylated within minutes, terminating signaling and reverting to active Myt1 and inactive Cdc25 levels [32]. The authors predict that a phosphatase is responsible for this threshold-sensing mechanism, but it has yet to be identified. Interestingly, inhibiting Cdk1/Cyclin B prevents this signal termination at sub-threshold levels of 1-MeAde, indicating that Cdk1/Cyclin B also functions as a negative regulator of its own activation in certain contexts.

### 7.3. Stick Insects (Clitarchus hookeri)

Stick insects have two paralogs of Wee1, referred to as Wee1a and Wee1b, which arose from a duplication event that is unique to *C. hookeri* and not conserved in other stick insect species [31]. Wee1a expression is restricted to gonad tissue of male and female stick insects and is much more highly expressed in males compared to females [31].

The stick insect *C. hookeri* can reproduce via parthenogenesis as an alternative to sexual reproduction. During parthenogenesis, an unfertilized oocyte can develop into an embryo without the requirement of fertilization by sperm. While meiosis must be altered for parthenogenesis to evolve, little is known about these requirements. Wu and colleagues annotated the transcriptomes of male and female stick insect gonads to look for changes in meiotic genes. Wee1 was identified as one of a group of meiotic genes that have undergone recent duplication events in stick insects [31]. This duplication of meiotic genes has also been observed in parthenogenic rotifers [105]. While the precise functions of Wee1a and Wee1b in *C. hookeri* meiosis remain to be elucidated, it is likely that changes in meiotic genes allow for greater flexibility in alternative reproduction methods such as parthenogenesis.

## 8. Discussion

### 8.1. General Summary

Wee/Myt family kinases are critical regulators of the meiotic cell cycle. By inhibiting the Cdk1/Cyclin B complex, Wee/Myt kinases prevent premature meiotic entry (Figure 1). The timing of meiotic entry differs greatly between spermatogenesis, which typically proceeds without arrest, and oogenesis, which typically involves long arrest periods. Wee/Myt kinases play sexually dimorphic roles to regulate meiotic entry in oogenesis versus spermatogenesis. In several organisms that have multiple Wee family kinases, Myt1 and/or Wee2 specifically regulate oogenesis, while Wee1 typically regulates spermatogenesis (Table 2). In animals that only have one Wee kinase active during meiosis, such as *C. elegans* or *Drosophila*, the kinase is differentially regulated, although the precise mechanisms remain unclear.

### 8.2. Evolution of Wee/Myt Kinases

**Figure 2:**
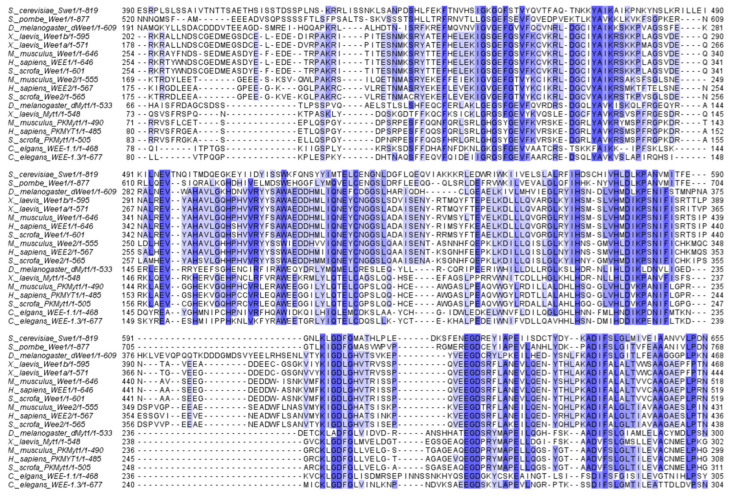
Conservation of Wee/Myt kinase domains. Multiple sequence alignment of Wee/Myt family kinases cropped to show conservation in the kinase domain. Sequences were aligned using the Clustal Omega algorithm [106]. Alignment was processed using Jalview version 2.11.5.1 [107]. Highlights show percent identity (darker purple indicates higher percent identity and lighter purple indicates lower percent identity).

**Figure 3:**
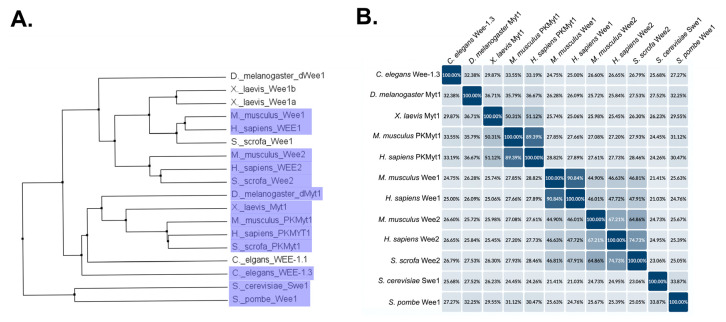
Overall conservation among Wee/Myt kinases. (**A**) Average distance tree of Wee/Myt family kinases created using Jalview [107]. Wee/Myt kinases known to play a role in meiosis are indicated by purple boxes. (**B**) Percent identity matrix for meiotic Wee/Myt family kinases created using Uniprot Align. Darker shading indicates a higher percent identity.

Wee/Myt family kinases for each species in this review are summarized in Table 1. A multiple sequence alignment of Wee/Myt kinase family members shows that the kinase domain is strongly conserved across species (Figure 2). To visualize the relationships between Wee/Myt family members across species, we created an average distance tree based on the multiple sequence alignment (Figure 3A). Aside from yeast Swe1 and Wee1, two distinct clades are present, which could be generalized as Wee-type kinases and Myt-type kinases. While the *C. elegans* orthologs are named WEE, they are dual kinases that phosphorylate both Thr 14 and Tyr 15 of Cdk1, making them more similar to Myt kinases than Wee kinases, which monophosphorylate Cdk1. Generally, Myt-type kinases regulate meiosis [28,36,51,55,71]. In mammals, Wee2 has emerged as an oocyte-specific Wee kinase [36,38,40], while Wee1 specifically regulates spermatogenesis in humans and mice [37,39,103]. Outside of the kinase domain, conservation varies extensively among the different species. Total percent identities are shown in Figure 3B. While some Wee/Myt family kinases discussed in this review have other known regulatory domains, they are poorly conserved. These regions are usually referred to as the N-terminal domain and the C-terminal domain. In general, these domains regulate Wee/Myt activity through species-specific mechanisms. Some examples include the N- and C-terminal regulatory regions of *Xenopus* Myt1, the C-terminal domain of *C. elegans* WEE-1.3 which regulates spermatogenesis, and the nuclear localization and nuclear export signals which govern Wee2 regulation in mammalian oocytes [36,43,53,74]. Future research could investigate how these domains regulate the meiotic activity of Wee/Myt kinases.

### 8.3. Wee/Myt Kinases in Oogenesis

While the basic mechanism of Wee/Myt kinase regulation during oogenesis is known, many questions remain. One of the challenges of studying Wee/Myt kinases during oogenesis is that Wee/Myt is required at multiple stages of oocyte meiosis. First, Wee/Myt kinase inhibition of Cdk1 coupled with continual Cyclin B degradation maintains oocyte arrest prior to meiosis I. Wee/Myt inhibition must be overcome for the oocyte to complete meiosis I. Then, Wee/Myt kinase inhibits Cdk1/Cyclin B again to prevent premature entry into meiosis II. Studies in mice have demonstrated that the mechanism of Wee/Myt activation differs between meiosis I and meiosis II [93]. More recent advances in conditional protein depletion techniques might be employed to dissect out meiosis I vs. meiosis II roles for Wee kinases.

### 8.4. Wee Kinases in Spermatogenesis

In general, Wee/Myt kinases have been more broadly studied during oogenesis, not spermatogenesis (Table 2). This is due in part to the relative ease of studying oocytes compared to spermatocytes in model organisms including *C. elegans* and *Xenopus*. While studies of Wee/Myt kinases in oogenesis have greatly contributed to the field’s fundamental understanding of Wee/Myt regulation, spermatogenesis differs greatly from oogenesis, and these different types of meiosis do not regulate meiotic entry in the same way. Unlike oogenesis, where many species have a Wee/Myt kinase that specifically regulates oocyte meiosis, spermatogenesis is generally regulated by Wee1, which is also required for mitosis [108]. Because of this, separating out the meiosis-specific mechanisms of Wee1 is challenging. Advances in techniques, including conditional protein depletion and tissue-specific knockdown, will be necessary to separate out the mitotic vs. meiotic roles of Wee kinases.

One major difference between oogenesis and spermatogenesis is that while oocytes generally lack centrioles, spermatocytes rely on centrioles for centrosome-based spindle assembly. Centrioles must duplicate twice during sperm meiosis, once during the premeiotic S phase and again between anaphase I and metaphase II. This second duplication is uncoupled from typical regulation which occurs during the S phase and only occurs in spermatocytes, making this process a key target of spermatogenesis-specific regulation. In yeast mitosis, Wee1 is known to regulate the spindle pole bodies (yeast centrosome equivalent) [109]. In *Drosophila*, knockdown of dMyt1 causes centrosome overduplication in spermatocytes [28]. Whether Wee1 regulates centrosomes in other species remains to be determined.

### 8.5. Regulatory Mechanisms of Wee/Myt Kinases

The most well-characterized role for Wee/Myt kinases is the phosphorylation of Cdk1 in the Cdk1/Cyclin B complex. While this mechanism is required for mitosis, spermatogenesis, and oogenesis, the downstream effects of Cdk1/Cyclin B vary depending on the type of cell division. Many species have multiple B type cyclins. Emerging evidence in *C. elegans* supports a model in which the specific Cyclin B in the complex triggers specific downstream effects [49,110,111,112]. This may be a typical mechanism for controlling specific M phase processes in different cell types. Cdk1/Cyclin B composition should be investigated in other species with multiple B type cyclins.

While Wee kinases are known to phosphorylate Cdk1 and Cdk2, beyond this only a few other substrates for Wee kinases have been identified in macrophages and cardiomyocytes [113,114]. Beyond Cdk1 and Cdk2, no other meiotic substrates of Wee kinases have been confirmed. Given that Wee kinases play distinct roles in meiosis compared to mitosis, it is possible that Wee kinases have meiosis-specific substrates. Kinase-substrate prediction tools and phosphoproteome analysis could be employed to identify potential target substrates for experimental validation.

Apart from kinase activity, studies in multiple species have identified meiotic regulatory regions in Wee family members [43,53,74]. These mechanisms play roles in sexually dimorphic Wee regulation during meiosis and may be functionally conserved in other species. It is likely that these non-kinase activities are responsible for some of the unknown aspects of Wee regulation and are a promising direction for future research.

### 8.6. Wee/Myt Kinases as Targets for Contraception

Examining the meiosis-specific roles of Wee/Myt kinases has revealed medically relevant insights. In mammals including humans, Wee2 kinase regulates oogenesis and is necessary for successful meiotic entry. The indispensable nature of Wee2 in mammalian oogenesis makes this kinase a promising target for non-hormonal contraceptives. Preliminary studies have identified Wee2 inhibitors which will be further evaluated [101].

Though less studied, Wee1 may also be a promising target for male contraception. This could potentially include Wee1 inhibitors, as a prior study found downregulation of Wee1 and its interactors to be associated with spermatogenesis failure in some patients [39]. A potential drawback to this approach is the risk of interfering with Wee1 regulation of mitosis. Rather than targeting Wee1 directly, interactors that are crucial to Wee1 regulation of spermatogenesis may also be promising targets. Such targets could be Cdc14a or Cdk2, as mutations in either of these genes causes male infertility in mice [86,87].

## 9. Conclusions

Wee/Myt kinases have long been known to be critical regulators of cell cycle progression. They play key roles in the G2/M phase transition. Historically, most of the focus has been on the role and regulation of Wee/Myt kinases in somatic cells undergoing mitosis. This review highlights the known functions of the Wee/Myt in the specialized cell division of meiosis in several different organisms. The investigations to date show that while some aspects of Wee/Myt function are conserved between cell types and organisms, there are several important aspects where Wee/Myt are involved in meiosis-specific functions or are regulated in a meiosis-specific way. Increased understanding of these crucial kinases in model systems will build our understanding of molecular mechanisms that drive the cell cycle and inform clinical treatments.

## Figures and Tables

**Table 2:** Summary of known meiotic roles of Wee kinase family members in indicated species.

Species	Wee Kinases Involved in Oogenesis	Wee Kinases Involved in Spermatogenesis	Role in Fertility
*D. melanogaster*	dMyt1	dMyt1	Loss of dMyt1 associated with fertility defects in spermatogenesis [28] and oogenesis [55]
*C. elegans*	WEE-1.3	WEE-1.3	WEE-1.3 required for spermatogenesis and oogenesis [43,51]
*C. hookeri*	Wee1a	Wee1a	Unknown
*A. pectinifera*	Myt1	Unknown	Myt1 required for oocyte meiosis [32]
*X. laevis*	Myt1	Unknown	Myt1 required for oocyte meiosis [71]
*A. testudineus*	Myt1	Unknown	Unknown
*Mus musculus*	PKMyt1, Wee2	Wee1	Wee1 regulates spermatogenesis [37] Wee2 required for oogenesis [93]
*S. scrofa*	Wee1b	Unknown	Wee1b required for oocyte meiosis [38]
*Homo sapiens*	PKMyt1, Wee2	Wee1	Loss of Wee1 associated with spermatogenesis defects [39] Loss of Wee2 associated with oogenesis defects [40,95,98,99,100]

## Data Availability

The original contributions presented in this study are included in the article. Further inquiries can be directed to the corresponding author.

## Abbreviations

The following abbreviations are used in this manuscript:

1-MeAde	1-methyladenine
APC/C	Anaphase promoting complex/cyclosome
CAK	CDK-activating kinase
CDK	Cyclin-dependent kinase
CRD	C-terminal regulatory domain
G2/M	G2-to-M phase transition
GFP	Green fluorescent protein
GVBD	Germinal vesicle breakdown
HU	Hydroxyurea
KD	Kinase domain
MIH	Maturation-inducing hormone
MPF	Maturation-promoting factor (Cdk1/Cyclin B)
NDJ	Nondisjunction
NES	Nuclear export signal
NEBD	Nuclear envelope breakdown
NLS	Nuclear localization signal
NRD	N-terminal regulatory domain
PKA	Protein kinase A
